# Implant Survival and Autologous Breast Augmentation in Rupture Management

**DOI:** 10.1093/asjof/ojag077

**Published:** 2026-05-05

**Authors:** Tung Dinh Nguyen

## Abstract

**Background:**

Silicone breast implants remain a mainstay of aesthetic surgery; however, long-term rupture remains a significant complication. MRI offers superior sensitivity for detecting silent ruptures. In patients declining reimplantation, autologous breast augmentation using a pedicled transverse rectus abdominis myocutaneous (TRAM) flap combined with fat grafting presents a natural, durable alternative.

**Objectives:**

The purpose of this study was to evaluate long-term implant survival and the outcomes of hybrid autologous augmentation using pedicled TRAM flap and fat grafting following explantation for rupture.

**Methods:**

A retrospective review was conducted on 150 female patients with 179 ruptured implants (June 2022-September 2024). Implant rupture was confirmed through 3T MRI. Implant longevity was analyzed using Kaplan–Meier survival. A subgroup of 41 patients underwent autologous reconstruction using a modified pedicled TRAM flap plus fat grafting. Intraoperative flap volume was calculated using a geometric model for truncated pyramids. Fat retention was quantified through MRI at 6 to 12 months. Patient-reported satisfaction was assessed using the BREAST-Q Postoperative Augmentation Module.

**Results:**

MRI demonstrated linguine (25.1%), teardrop (15.6%), and combined rupture signs. Kaplan–Meier analysis showed a 20-year implant survival rate of 83%. In the autologous group (*n* = 41), the average intraoperative volume was 328.5 cc, with 83.0% fat retention on follow-up MRI. The mean BREAST-Q satisfaction score was 62.7, positively correlating with volume retention (*r* = 0.317, *P* = .043).

**Conclusions:**

Hybrid autologous breast augmentation using a TRAM flap and fat grafting is a reliable and aesthetically satisfying alternative to reimplantation. MRI facilitates accurate rupture diagnosis and postoperative volumetric monitoring. This technique also yields abdominal contouring benefits and reduced long-term costs, making it an attractive option for selected patients.

**Level of Evidence: 4 (Therapeutic):**

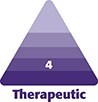

Breast augmentation remains the most commonly performed cosmetic surgery worldwide, with over 1.6 million procedures reported globally in 2022, of which ∼85% utilized silicone implants.^1,2^ Despite continuous improvements in implant design and manufacturing, complications such as rupture persist, particularly over long-term follow-up.^2^ Studies have shown that the cumulative rupture rate increases with implant age, with reports suggesting 10% to 15% rupture by Year 10 and 25% to 35% by Year 20.^2,3^

Silicone implant rupture may be intracapsular or extracapsular and is frequently asymptomatic. Silent ruptures account for 60% to 80% of all cases and are often undetected by physical examination alone.^4^ Although high-definition ultrasound is increasingly employed because of accessibility and cost advantages, its sensitivity for silent implant rupture remains lower (55%-70%) compared with MRI (>90%).^5,6^ In this context, MRI has emerged as the most accurate diagnostic modality, with sensitivity and specificity exceeding 90%.^5,6^ The US FDA recommends MRI screening 5 to 6 years postimplantation, then every 2 years thereafter.^4^ Diagnostic signs include the linguine sign (collapsed shell), teardrop sign (gel bleed), keyhole sign (intracapsular herniation), and subcapsular line (extracapsular spread); therefore, our institution continues to use MRI for rupture surveillance and particularly as MRI also permits volumetric analysis.^5,6^

The psychological impact of rupture is considerable. Surveys indicate up to 42% of patients report anxiety or body image concerns upon learning of a rupture. The growing awareness of breast implant illness and rare occurrences of breast implant-associated anaplastic large cell lymphoma (BIA-ALCL) has contributed to rising explantation rates.^2^ Approximately 20% to 30% of explantation patients now decline reimplantation, opting instead for natural and durable alternatives.

Currently, surgical options after implant removal because of rupture include: (1) simple explantation with or without capsulectomy; (2) implant replacement with or without site change (subglandular to submuscular); (3) total capsulectomy with fat grafting alone; and (4) autologous tissue reconstruction using local or distant flaps such as deep inferior epigastric perforator (DIEP), superior gluteal artery perforator (SGAP), or transverse rectus abdominis myocutaneous (TRAM) flaps.^7,8^ Among these, implant replacement remains common but is associated with repeat rupture risks, whereas fat grafting alone often requires multiple sessions to achieve durable volume. Hybrid techniques using flaps and fat grafting aim to combine reliable volume, projection, and long-term aesthetic outcomes.

In response to this shift, an increasing number of patients seek autologous reconstruction following implant removal. The pedicled TRAM flap remains a reliable technique, offering vascularized soft tissue and volume replacement.^7,9^ However, traditional TRAM flaps often yield inadequate contour and projection, particularly in slim patients or bilateral cases.

This study introduces a modified TRAM technique—high-level muscle dissection with neurectomy and partial skin paddle transposition into a prepectoral pocket—which improves conformity and upper pole fullness and reduces inframammary bulging.^7,9,10^

Autologous fat grafting is widely used in aesthetic breast surgery for contour refinement, correction of contour deformities, and enhancement of breast shape. Coleman described standardized techniques for breast augmentation using structural fat grafting, emphasizing meticulous harvesting, processing, and multilayer injection to optimize graft survival.^8^ It allows refinement of contour and cleavage, with volume retention is influenced by grafting technique, recipient-site vascularity, and postoperative biological remodeling.^11-13^

## METHODS

### Patient Selection

This retrospective study included 150 female patients who experienced a total of 179 breast implant ruptures between June 2022 and September 2024. All procedures were performed at EMCAS Plastic Surgery Hospital (Ho Chi Minh City, Vietnam), a high-volume aesthetic practice with academic affiliations. This setting provides comprehensive perioperative monitoring and postoperative care, enabling complex autologous reconstructions to be performed safely in carefully selected elective patients.

All patients had previously undergone cosmetic breast augmentation and suggested rupture in all cases by 3T MRI. Importantly, this study did not include patients with intact implants or those without rupture and therefore does not represent the overall community of implant recipients. Rather, it focuses specifically on outcomes following rupture.

Inclusion criteria were age 18 to 65 years, previous silicone breast implants with MRI-confirmed rupture, BMI <30, no personal or familial history of breast cancer, no history of radiation therapy, nonsmoking status or cessation for at least 6 months, and no plans for future pregnancy. Patients with active infections, uncontrolled comorbidities, or psychiatric instability were excluded.

Among this cohort, 41 patients elected to undergo hybrid autologous breast augmentation using a pedicled TRAM flap combined with fat grafting, which defines the subgroup analyzed for reconstructive outcomes.

Of the 150 patients with MRI-confirmed implant rupture, 63 declined reimplantation and were screened for autologous reconstruction. Exclusion criteria included insufficient abdominal donor tissue (*n* = 12), high BMI with elevated perioperative risk (*n* = 4), previous abdominal surgery compromising flap harvest (*n* = 3), comorbidities or age-related anesthetic risk (*n* = 2), and refusal of hospitalization/recovery despite eligibility (*n* = 1). The remaining 41 patients underwent pedicled TRAM flap with fat grafting. A CONSORT-style flowchart figure summarizes patient inclusion and exclusion.

This study was conducted in accordance with the ethical principles of the Declaration of Helsinki. Ethical approval was obtained from the IRB of EMCAS Plastic Surgery Hospital, Ho Chi Minh City, Vietnam (IRB approval no. 156/QD-EMCAS dated May 10, 2025). All patients provided written informed consent before participation, including consent for surgical procedures, photography, and the use of anonymized clinical data for research and publication purposes.

### Flowchart Figure

- Assessed for eligibility: *n* = 150
- Declined reimplantation: *n* = 63
  - Excluded: *n* = 22
    - Low BMI/inadequate donor tissue (*n* = 12)
    - High BMI/elevated risk (*n* = 4)
    - Previous abdominal surgery (*n* = 3)
    - Age/comorbidity risk (*n* = 2)
    - Declined hospitalization (*n* = 1)
  - Included: TRAM + fat grafting (*n* = 41)

### Diagnostic Evaluation

Although high-resolution ultrasound is commonly used in the United States, our institution follows the FDA's guidance for MRI surveillance beginning 5 to 6 years after implantation and every 2 years thereafter, because of MRI's superior sensitivity (94% vs 55%) in detecting silent rupture.^5,6^ Clinical examination and 3T MRI were performed in all cases to assess implant integrity and rupture features. MRI protocols included silicone-specific sequences using dedicated breast coils, focusing on rupture indicators such as linguine sign, teardrop sign, keyhole deformity, and extracapsular silicone leakage. High-resolution ultrasound was not routinely used, in accordance with FDA guidelines recommending MRI as the gold standard for silent rupture evaluation.^4-7^

### Implant Survival Analysis

Kaplan–Meier survival analysis was conducted to evaluate long-term implant integrity. Survival curves were generated based on the date of initial implantation and the MRI-suggested rupture date, allowing comparison of rupture rates at 10 and 20 years postimplantation.^3,9,14^ Kaplan–Meier survival analysis defined rupture as the event. The 12 implants found intact at explantation were treated as right-censored cases. Postoperative volumetric MRI assessment was performed only in the 41 patients who underwent hybrid TRAM+ fat grafting reconstruction.

### Surgical Technique

Of the 150 patients, 41 who declined implant replacement underwent autologous breast augmentation using a modified TRAM flap combined with fat grafting. All procedures were performed by a single senior plastic surgeon under general anesthesia.

After explantation and complete capsulectomy, the ruptured implant was removed through a superior periareolar incision. In patients with breast ptosis secondary to long-standing implants, a crescent of periareolar skin was excised, allowing mastopexy and elevation of the breast parenchyma to correct sagging.

A pedicled TRAM flap was harvested based on the superior epigastric vessels, which were clearly visualized during dissection. The flap was then divided into 2 segments, each designated for augmentation of one breast pocket.^13,15^

The entire skin paddle was fully de-epithelialized to maximize the usable flap volume. During harvest, intercostal nerve branches T7 to T12 were identified and transected at the medial rectus border to achieve denervation and improve pliability.

For inset, the flap was elevated superiorly with selective thinning at the inframammary fold to facilitate turnover into the breast pocket and reduce bulk. Each flap half was inset into the prepared breast pocket and supplemented with autologous fat grafting for contour refinement and symmetry.^10,16^

At the donor site, the abdominal wall defect was reconstructed with dual-layer lightweight polypropylene mesh: an inlay bridging the fascial defect and an onlay reinforcing the anterior rectus sheath. This reinforcement minimized the risk of bulging or hernia.

Key procedural steps are demonstrated in Video which provide intraoperative detail with synchronized narration, embedded captions, and visual highlights. Video demonstrates the operative sequence, including complete flap de-epithelialization, pedicle preservation, nerve transection, flap tunneling, fat graft harvest and injection, and inset into the breast pocket. Key steps are annotated to highlight the modifications of the pedicled TRAM technique for autologous breast augmentation.

### Fat Grafting Protocol

Simultaneously, autologous fat was harvested from the abdomen, flanks, or thighs using a low-pressure aspiration system. The fat was centrifuged at 3000 rpm for 3 min and injected in small aliquots (<0.1 mL per pass) into the medial and upper pole subcutaneous and intramuscular planes. Fat grafting was used to refine symmetry and cleavage, especially in patients with thinner soft tissue coverage.^12,17^

### Fat Volume Assessment

**Figure 1.**
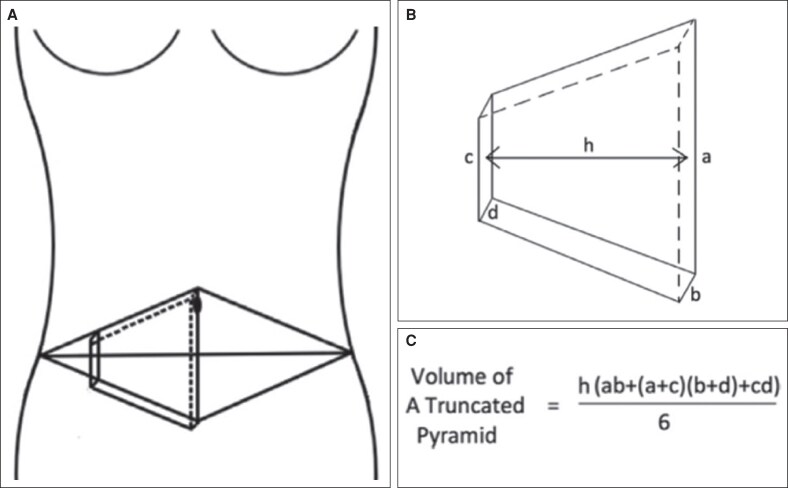
Geometric estimation of intraoperative TRAM flap volume using a truncated pyramid model. (A) Anatomical location and design of the pedicled TRAM flap. (B) Geometric interpretation of flap boundaries, modeled as a truncated pyramid. (C) Mathematical formula used to calculate intraoperative flap volume. Illustration created by the author.

Intraoperative volume (*V*1) was calculated based on geometrical modeling of the TRAM flap as a truncated pyramid, as illustrated in Figure 1. Measurements were taken intraoperatively for the base width (a), base length (b), top width (c), top length (d), and vertical height (h) of the flap, as defined in the diagram. The volume was then computed using the formula, as described by Razzano et al:^18^

V1=[h×(ab+(a+c)(b+d)+cd)]/6

Fat graft volume was added to the calculated TRAM flap volume to derive total intraoperative volume (*V*1). Postoperative volume (*V*2) was measured using 3T MRI at 6 to 12 months after surgery. Breast volume was segmented and calculated using dedicated imaging software, and fat retention was calculated as:

Fatretention(%)=(V2/V1)×100

### Donor-Site Management

All donor sites were reinforced with polypropylene mesh to reduce abdominal. Donor-site morbidity was prospectively assessed during routine follow-up at 1, 3, 6, and 12 months. Abdominal wall weakness was defined by both clinical examination (manual resistance testing of trunk flexion and straight-leg raise) and patient-reported functional symptoms. All patients underwent mesh reinforcement of the abdominal closure to minimize bulging or hernia formation.

### Patient Satisfaction Assessment

All patients completed the BREAST-Q Augmentation Module (Postoperative Version 2.0) at 6 to 12 months after surgery. The Rasch-transformed scores were used to assess satisfaction with breast aesthetics, psychosocial well-being, and physical comfort. Correlations between volume retention and satisfaction were assessed using Pearson's *r* correlation.^17^

### Statistical Analysis

All data were collected prospectively and analyzed using IBM SPSS Statistics version 20 (IBM Corp., Armonk, NY). Given the sample size, only descriptive and bivariate statistics (eg, Pearson's correlation) were applied; multivariate regression was not performed to avoid model overfitting. Statistical significance was set at *P* < .05.

## RESULTS

This study investigated rupture management strategies in a cohort of 150 women with a total of 179 ruptured breast implants between June 2022 and September 2024. Of the 179 explanted implants, 165 (92.2%) were silicone gel–based, and 14 (7.8%) were saline filled. A total of 151 implants (84.4%) had textured surfaces, whereas 28 (15.6%) had smooth surfaces. Among the 41 patients who underwent autologous reconstruction with pedicled TRAM flap and fat grafting, implant position data were available: 29 implants (70.7%) were placed in the subglandular plane and 12 (29.3%) in the submuscular plane. These patients were primarily healthy individuals without oncologic histories or plans for future pregnancy, highlighting the relevance of this hybrid approach in elective, aesthetic revision settings.

**Figure 2.**
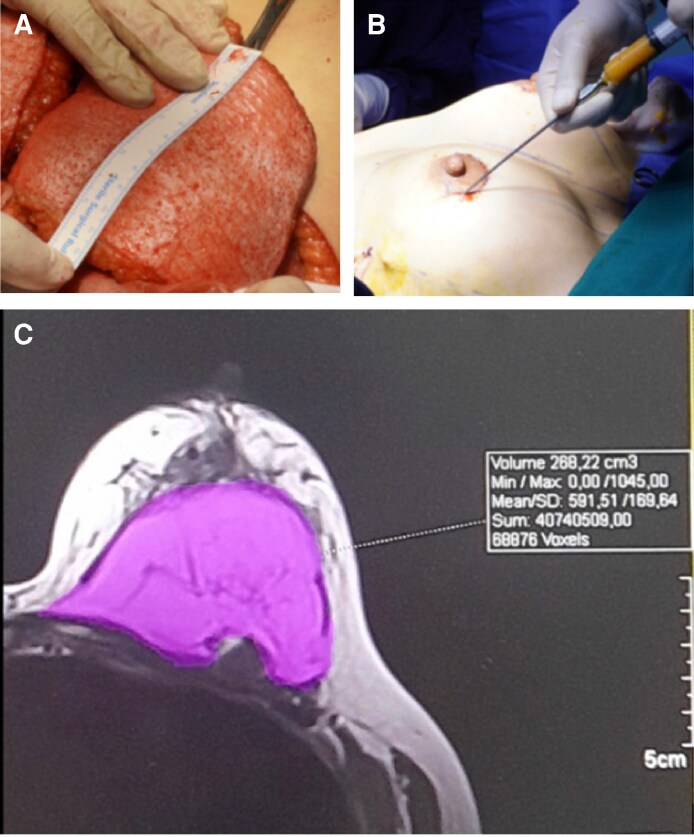
Volumetric assessment in autologous breast augmentation. Intraoperative evaluation of total breast volume using pedicled TRAM flap measurement—(A) VTRAM and (B) fat grafting with a multi-port cannula. (C) Postoperative MRI volumetry at 6 months demonstrating segmented retained volume of 268.22 cm^3^. TRAM, transverse rectus abdominis myocutaneous; VTRAM, volume of the transverse rectus abdominis myocutaneous flap.

**Figure 3.**
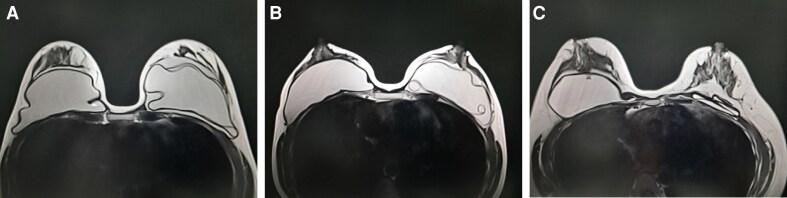
(A) MRI findings of intracapsular rupture showing linguine sign. (B) MRI findings of intracapsular rupture showing teardrop sign. (C) MRI findings showing extracapsular rupture.

MRI played a critical role in preoperative evaluation, suggested rupture in all 179 implants and demonstrating both intracapsular and extracapsular rupture patterns. MRI findings included classic rupture signs such as the linguine sign in 25.1% of cases and extracapsular rupture in 8.9% (Figures 2, 3).

At surgery, rupture was confirmed in 167 implants (93.3%), whereas 12 implants (6.7%) were intact. For Kaplan–Meier analysis, intraoperatively confirmed rupture was the event, and the 12 intact implants were censored at explantation. We emphasize that MRI's role was diagnostic and volumetric, whereas the Kaplan–Meier analysis was based on surgical confirmation. This ensures there is no overlap or discrepancy between imaging findings and survival analysis.

**Figure 4.**
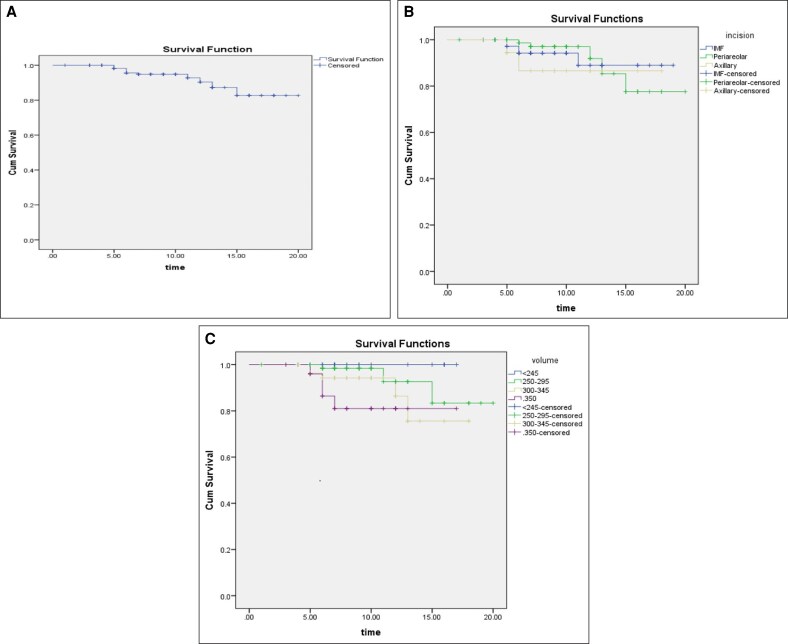
(A) Kaplan–Meier survival analysis of breast implants over 20 years. The cumulative survival probability was 95% at 10 years and 83% at 20 years. Since only one group was analyzed, no log-rank *P*-value is applicable. Censored cases are indicated by cross marks. (B) Kaplan–Meier comparison of implant survival stratified by incision type (inframammary, periareolar, and axillary). Implants inserted through the inframammary fold (IMF) showed significantly longer survival compared with periareolar and axillary incisions (log-rank *P* = .02). Censored cases are indicated by cross marks. (C) Kaplan–Meier survival analysis stratified by implant volume. Implants <245 mL demonstrated significantly higher long-term survival compared with implants ≥245 mL (log-rank *P* = .01). Censored cases are indicated by cross marks.

Using this method, implant survival was estimated at 95% at 10 years and 83% at 20 years. (Figure 4A). Subanalysis indicated that inframammary incisions and smaller implant volumes (<245 mL) were associated with longer survival (Figure 4B, C).

Most of the explanted implants were silicone gel–based and had textured surfaces, with a majority placed in the subglandular plane. These characteristics may have contributed to the observed rupture profiles and inform future implant selection and placement decisions.

The mean age at primary breast augmentation was 31.41 years, with an estimated age range of 19 to 43 years. The average time to implant rupture was 8.79 years (standard deviation = 3.74). The mean age at rupture of 40.2 years, with an estimated range of ∼33.7 to 48.9 years (Table 1).

**Table 1.** Implant Rupture Characteristics and Imaging Findings (*n* = 179)

Characteristic	Category	Frequency (*n*)	%
Mean age (at augmentation timing)		31.41 years	Range:19.0-43.0
The average time to implant rupture		8.79 years	(SD = 3.74)
Mean age (at rupture)		40.2 years	Range:33.7-48.9
Implant rupture side	Right	84	46.9%
	Left	95	53.1%
Capsular contracture	Yes	53	29.6%
	No	126	70.4%
Incision type	Inframammary fold	76	42.5%
	Periareolar	84	46.9%
	Axillary	19	10.6%
Implant volume (mL)	<245	22	12.3%
	250-295	72	40.2%
	300-345	59	33.0%
	>350	26	14.5%
Clinical features	No complaint	82	45.8%
	Pain	44	24.6%
	Hardening	14	7.8%
	Deformity	19	10.6%
	Others	20	11.2%
MRI findings (rupture sign)	Linguine sign	45	25.1%
	Subcapsular Collection	27	15.1%
	Teardrop sign	28	15.6%
	Keyhole sign	26	14.5%
	Extracapsular Rupture	16	8.9%
	Combined signs	37	20.7%
Postrupture intervention	Explantation only	22	12.3%
	Reimplantation	66	36.9%
	Implant + fat grafting	50	27.9%
	TRAM flap + fat grafting	41	22.9%

SD, standard deviation; TRAM, transverse rectus abdominis myocutaneous.

The majority of implants in this cohort were textured silicone gel devices from leading international manufacturers, placed in the subglandular plane. These characteristics reflect implant selection trends during the timeframe in which the original augmentations were performed.

### Implant Longevity

Kaplan–Meier survival showed implant survival rates of 95% at 10 years and 83% at 20 years. Inframammary incisions and implants <245 mL were associated with longer survival, whereas larger volumes and periareolar or axillary incisions had higher rupture rates.

## DISCUSSION

**Figure 5.**
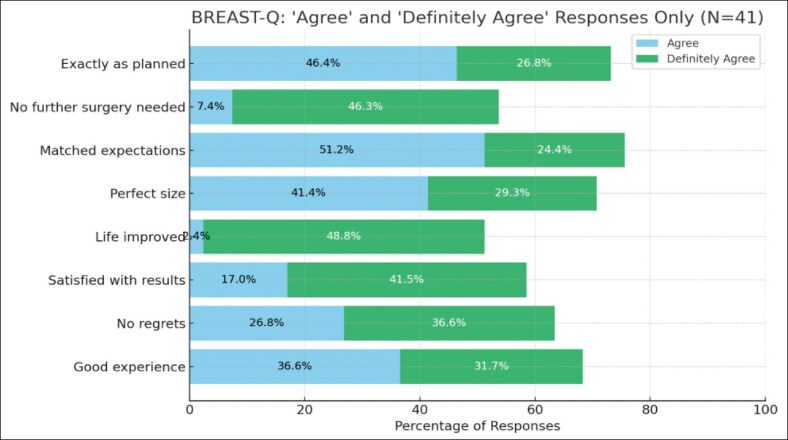
Distribution of BREAST-Q postoperative satisfaction scores. Patient-reported outcomes were evaluated using the BREAST-Q Augmentation Module (postoperative, v2.0). All 41 patients completed the questionnaire at 6 to 12 months postoperatively. The BREAST-Q consists of Rasch-transformed domains assessing satisfaction with breast aesthetics, psychosocial well-being, and physical comfort. The overall satisfaction score averaged 62.7. The mean BREAST-Q satisfaction score was 62.7, reflecting moderate-to-high satisfaction. Domain-specific analysis revealed highest scores in breast softness and natural feel, followed by improved confidence and body image. Lower scores were noted for breast symmetry and fullness. Patients with higher intraoperative volume (*V*1) tended to report better alignment with their expectations.

**Figure 6.**
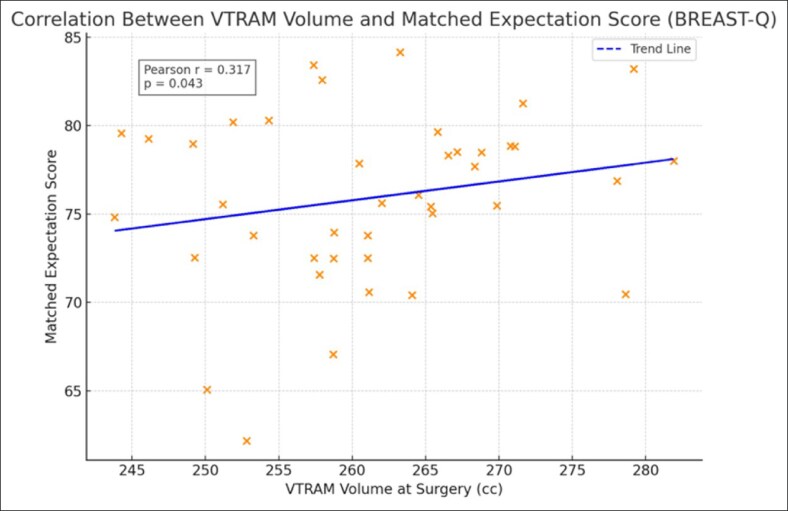
Scatter plot with linear regression line showing the correlation between VTRAM + injected fat graft volume and BREAST-Q satisfaction score. The regression line equation (*y* = 0.28*x* + 52.3) and coefficient of determination (*R*^2^ = 0.31) are displayed on the graph. Although a positive correlation was observed, it did not reach statistical significance (*P* = .08). VTRAM, volume of the transverse rectus abdominis myocutaneous flap.

This study offers a detailed analysis of surgical outcomes using autologous reconstruction with TRAM flap and fat grafting in the context of implant rupture. Implant characteristics—such as silicone gel composition, textured surfaces, and subglandular positioning—may have contributed to the rupture patterns observed. Although these associations are plausible, they should be interpreted cautiously, because our study design was not intended to determine causality. Nonetheless, these findings could inform future decision making regarding implant selection and placement.^1^ The results highlight the diagnostic value of MRI (Figure 4A-C, Table 1), the volumetric advantages of the hybrid approach (Table 2), and the patient satisfaction and cost-effectiveness benefits of avoiding prosthetic reimplantation (Figures 5, 6).

**Table 2.** Fat Volume Summary (*n* = 41)

Measurement	Mean volume/value
TRAM flap volume	263.4 cc
Fat grafting volume	65.1 cc
Total intraoperative volume (*V*1)	328.5 cc
Postoperative MRI volume (*V*2)	272.7 cc
Fat retention rate	83.0%

TRAM, transverse rectus abdominis myocutaneous.

Although high-definition ultrasound is evolving and offers a cost-effective screening tool, MRI provided superior diagnostic sensitivity and enabled quantitative volume assessments central to our fat retention analysis.^5,6^ This dual utility supports its continued role as the preferred modality in our practice reinforces MRI's role as the gold standard for rupture diagnosis.^4-6^ This high accuracy enabled precise surgical planning and appropriate patient selection. MRI findings in this study were consistent with previous research, such as the linguine sign rate of 25.1% and the extracapsular rupture rate of 8.9%.^6^

**Figure 7.**
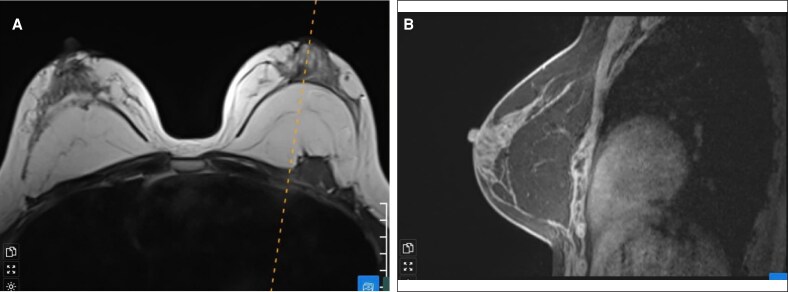
The patient, a 38-year-old woman, underwent explantation because of right-sided implant rupture. (A) Postoperative MRI at 12 months following bilateral implant removal and autologous breast augmentation with transverse rectus abdominis myocutaneous (TRAM) flap and fat grafting. Axial MRI shows well-integrated TRAM flap volume with symmetric fat distribution in both breasts. (B) Postoperative MRI at 12 months following bilateral implant removal and autologous breast augmentation with TRAM flap and fat grafting. Sagittal MRI reveals stable fat graft retention and soft-tissue volume without evidence of oil cysts, seroma, or fat necrosis.

It is important to clarify that Kaplan–Meier survival curves were generated from the entire rupture cohort (*n* = 179 implants), with intraoperative confirmation of rupture defined as the event. The 41 patients who underwent TRAM + fat grafting belonged to this same rupture cohort, but postoperative MRI was performed only in this subgroup for volumetric assessment. Thus, MRI served as both a diagnostic tool in the rupture cohort and a volumetric monitoring tool in the reconstruction subgroup, without overlap between diagnostic and survival endpoints (Figure 7A, B).

In our cohort, among 179 cases with imaging signs suggestive of implant rupture, 12 (≈6.7%) were found intact at surgery. This imaging false-positive rate is relatively modest compared with previous reports in the literature: for example, Zingaretti et al observed a ∼33% rate of intact implants among explanted prostheses originally suspected of rupture, whereas Lindenblatt et al found only 41% concordance between MRI positivity and surgical rupture (ie, 59% false positives).^19,20^ The phenomenon of radiologic overdiagnosis is well documented, with potential contributors including interpretative artifacts (eg, “rat-tail” sign) gel bleed mimicking rupture, interobserver variability.^21^ Our lower false-positive proportion may reflect stricter imaging criteria, higher radiologist expertise, or conservative thresholds for calling rupture. Nevertheless, our findings reinforce the need for cautious interpretation of imaging findings and the incorporation of clinical, imaging, and surgical correlation in decision making.

Kaplan–Meier analysis in our cohort demonstrated implant survival rates of 95% at 10 years and 83% at 20 years. These findings are consistent with previous longterm clinical studies showing that silicone breast implants generally demonstrate favorable durability during the first decade after augmentation, with rupture prevalence increasing progressively over time.^3^ Furthermore, the association between incision type and implant longevity observed in our study was in line with Brown et al, who found higher rupture incidence with periareolar and axillary incisions compared with inframammary access.^9^

Although implant rupture was unilateral in many cases, the majority of patients elected to remove both implants for reasons of symmetry and peace of mind. This patient-driven decision is consistent with previous reports highlighting the psychological and aesthetic considerations that strongly influence management choices following rupture.

The TRAM flap is known for its reliable vascularity and substantial donor volume. According to Nahabedian et al, TRAM flaps remain a gold standard in autologous breast reconstruction, with complication rates under 5% in well-selected patients.^7^ Our series reported no flap losses, reaffirming its safety profile in nononcologic indications.

Fat grafting was performed using the Coleman technique. According to Khouri et al.,^12^ successful fat graft survival depends on adequate recipient-site perfusion, microaliquot injection, and rapid revascularization of the transplanted adipose tissue. In our series, these principles resulted in a mean retention rate of 83.0% at 6–12 months. (Table 2) This retention rate is higher than that generally reported for autologous fat grafting performed without vascularized flap support. A systematic review by Groen et al.^11^ demonstrated considerable variability in volume retention, with outcomes influenced by surgical technique, recipient-site characteristics, imaging methodology, and duration of follow-up. Lin et al.^13^ reported 65% retention at 6 months using three-dimensional imaging, whereas the systematic review by Largo et al.^15^ demonstrated that fat graft retention and complication rates vary considerably according to surgical technique, recipient-site characteristics, graft processing, and duration of follow-up. Ørholt et al.^10^ further demonstrated that long-term volume retention after breast augmentation with fat grafting is influenced by postoperative weight changes, with MRI showing stabilization of graft volume during follow-up. Tissiani et al.16 reported nearly complete fat retention (~99.8%) in a TRAM flap recipient after 33 months, further supporting the favorable long-term volume preservation observed in our study.

This hybrid method reduces the need for multiple sessions and minimizes long-term patient cost and recovery time. Although the initial cost of TRAM + fat grafting is higher, its overall cost-effectiveness improves when accounting for the rarity of reoperations and absence of implant-related complications (Table 3).^22-24^

**Table 3.** Cost Comparison

Item	Modified TRAM + fat grafting (Vietnam)	implant replacement (Vietnam)	Modified TRAM + fat grafting (USA)^a^	implant replacement (USA)^a^
Initial surgical cost	$6000–$12,000 USD	$3000–$5000 USD	$20,000–$30,000 USD	$8000–$12,000 USD
Hospital stay	3-5 days	1 day (outpatient or overnight)	5-7 days	1 day
Reoperation frequency	Rare (one-time procedure)	Likely every 10-15 years	Rare	Common (revision at 10-15 years)
Implant cost	Not applicable	$1000–$2000 USD/pair	Not applicable	$2000–$3000 USD/pair
Long-term complication management	Minimal	May include rupture, capsular contracture	Minimal	As above
Aesthetic revision needs	Rare	20%-30% may need revision	Rare	Common
Additional benefit	Includes abdominoplasty	None	Includes abdominoplasty	None

TRAM, transverse rectus abdominis myocutaneous.

^a^US cost estimates are approximate and based on published literature and institutional billing data.^22,23^

Cost comparisons must be interpreted within the context of local healthcare economics. In our setting, autologous reconstruction with TRAM flap and fat grafting was cost-effective relative to reimplantation. However, in many healthcare systems, the cost of a bilateral TRAM flap with hospitalization and recovery may be substantially higher than implant replacement or removal. Thus, the economic implications of this approach are highly region specific.

It is important to note that the economic implications of this approach vary significantly by healthcare setting. In our center, the cost of autologous reconstruction using TRAM flap and fat grafting ranged from $6000 to $12,000 USD. However, in the United States, the cost of bilateral pedicled TRAM flap reconstruction has been reported to exceed $20,000 to $30,000 USD, excluding hospital stay and additional postoperative care.^22,23^ Implant-based revision with replacement may also cost $8000 to $15,000 USD depending on the complexity of revision and geographic region. Therefore, although our findings support the cost-effectiveness of this technique in our local context, extrapolation to Western healthcare systems must be made cautiously.

Additionally, this procedure provides the aesthetic and contouring benefits of simultaneous abdominoplasty, which may be valued by patients seeking both breast reconstruction and abdominal rejuvenation, particularly postpregnancy or postweight loss cases. TRAM flap also offers a robust scaffold for fat integration, enhancing volume stability and contour refinement.

BREAST-Q scores (Figure 5) indicated high satisfaction, particularly in breast softness, natural feel, and body image. The positive correlation between intraoperative volume and satisfaction score (*r* = 0.317, *P* = .043) is consistent with McCarthy et al, who emphasized volume and projection as key satisfaction determinants.^25^

Our patients reported a mean Rasch-transformed BREAST-Q score of 62.7, corresponding to moderate-to-high satisfaction. Although not in the uppermost satisfaction range, this score compares favorably with published reports of secondary breast reconstruction and underscores the importance of adequate postoperative volume in shaping patient-perceived outcomes (Figures 5, 6, 7A, B).

Complication rates were low, with no major adverse outcomes. Minor issues included wound dehiscence (2.4%), seroma (4.8%), transient abdominal wall weakness (4.8%), and fat necrosis (7.3%), as shown in Table 4. These rates are comparable or lower than those reported for implant-based surgeries.^22,23^

**Table 4.** Complication Comparion

Complication type	Specific complication	Modified TRAM + fat grafting (%)	Implant-based surgery (%)^22,23^
Early (≤30 days)	Minor wound dehiscence	2.4	3-5
	Donor-site seroma	4.8	1-2
	Abdominal wall weakness	4.8	NA
Late (>30 days)	Fat necrosis	7.3	NA
	Infection	0	1-3
	Hematoma	0	1-2
	Hernia	0	<1

NA, not available; TRAM, transverse rectus abdominis myocutaneous.

Our low incidence of transient abdominal wall weakness (4.8%)—all resolving by 6 months—contrasts with the higher morbidity historically reported for bilateral rectus harvest. This favorable outcome, together with the low rate of fat necrosis and high fat retention (83%), can be attributed to systematic mesh reinforcement, early mobilization, structured follow-up, meticulous flap harvest preserving vascularity, and micro-aliquot fat injection into the well-vascularized TRAM bed. These refinements likely explain the superior results compared with historical TRAM series (Figures 2, 7A, B).

**Figure 8.**
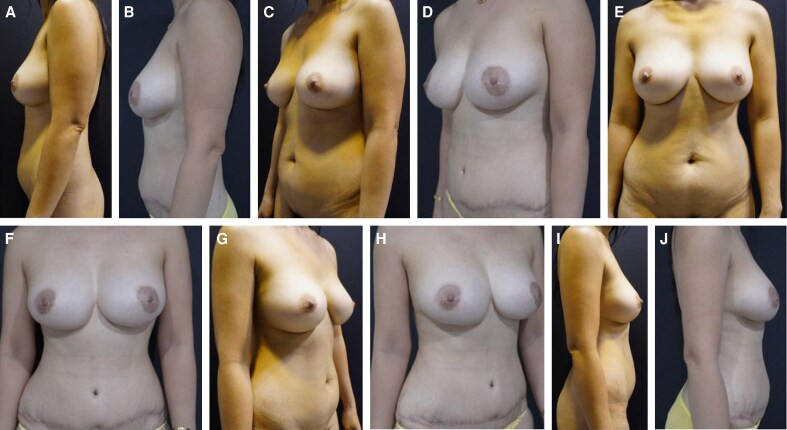
Clinical photographs before and after autologous breast augmentation in a 46-year-old patient. (A, C, E, G, I) Preoperative images before implant removal for right-sided rupture, 13 years after primary breast augmentation. (B, D, F, H, J) Postoperative appearance at 12 months following bilateral implant removal and autologous breast augmentation using pedicled transverse rectus abdominis myocutaneous flap and fat grafting.

**Figure 9.**
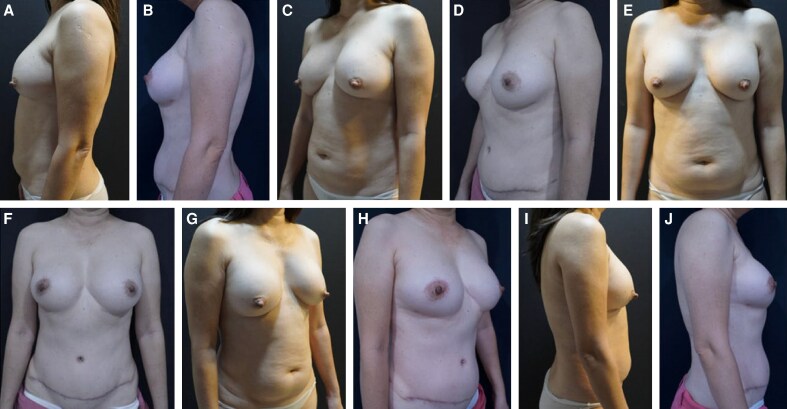
Clinical photographs before and after autologous breast augmentation in a 52-year-old patient. (A, C, E, G, I) Preoperative views demonstrating bilateral breast implant rupture, 18 years after primary augmentation. (B, D, F, H, J) Postoperative views at 12 months following bilateral explantation and autologous breast augmentation with pedicled transverse rectus abdominis myocutaneous flap and fat grafting.

Photographic documentation further reinforces the clinical relevance of this hybrid procedure in real patients (Figures 8, 9), illustrating aesthetic improvements and volume symmetry at follow-up.

Collectively, these findings reinforce the value of MRI in rupture detection, support the hybrid TRAM-fat grafting technique for selected patients, and highlight its cost-effective, high-satisfaction outcomes in implant failure management.

This technique is appropriate only for a carefully selected subgroup of patients who decline reimplantation, possess adequate donor tissue, and are motivated to accept the potential risks and recovery associated with a TRAM flap. It should be regarded as a patient driven, selective alternative performed in centers with comprehensive perioperative care, rather than a mainstream reconstructive option.

Our findings align with previous reports demonstrating the reliability of pedicled TRAM flaps for autologous breast reconstruction. However, unlike traditional TRAM series where fat necrosis rates range from 10% to 25% and donor-site weakness remains a concern, our rates of fat necrosis (4.8%) and transient abdominal weakness (4.8%, resolved by 6 months) compare favorably, likely reflecting systematic mesh reinforcement and meticulous technique. Similarly, our fat graft retention of 83% at 12 months is higher than many previously reported hybrid autologous approaches, where retention is often limited to 50% to 70%. These results underscore the potential of combining pedicled TRAM with adjunctive fat grafting to achieve durable volume and shape in patients undergoing implant removal.

This favorable outcome, together with the low rate of fat necrosis and high fat retention can be attributed to systematic mesh reinforcement, early mobilization, structured follow-up, meticulous flap harvest preserving vascularity, and micro-aliquot fat injection into the well-vascularized TRAM bed. These refinements likely explain the superior results compared with historical TRAM series.

Importantly, although patient satisfaction (mean BREAST-Q score 62.7) was moderate to high, it compares favorably with secondary implant-based reconstructions, supporting this approach as a viable alternative for motivated patients. Nevertheless, these benefits must be weighed against the longer recovery and donor-site morbidity inherent to TRAM flaps, limiting its role to a carefully selected subgroup.

### Limitations

Despite promising results, limitations exist. Our study has several limitations. First, it was a retrospective, single-center series conducted by a single surgeon, which may introduce selection bias and limit generalizability. Second, the sample size was relatively small (*n* = 41 in the TRAM + fat grafting group), precluding multivariate regression to adjust for potential confounders such as BMI, implant volume, and patient age. Third, follow-up was limited to 6 to 12 months, which may underestimate long-term complications, durability of fat graft retention, abdominal wall morbidity, and functional outcomes. Fourth, photographic documentation was not fully standardized in terms of lighting and positioning, potentially affecting the educational value of comparative images. Fifth, cost-effectiveness of the hybrid TRAM and fat grafting technique was not formally analyzed; moreover, the pricing structure at our center—where operating costs are lower and bundled surgical care is common—does not reflect typical expenses in North American or Western European healthcare systems. Therefore, extrapolation of cost feasibility to international contexts should be interpreted with caution. Larger, multicenter prospective studies with standardized assessment tools and longer follow-up are needed to validate the reproducibility, durability, and generalizability of these findings.

## CONCLUSIONS

This study demonstrates that autologous breast augmentation using a pedicled TRAM flap combined with fat grafting may be a safe, reliable, and satisfactory option for patients undergoing implant removal following rupture. The technique offers a high fat retention rate (83.0%) and an acceptable complication profile, with no major adverse outcomes reported. MRI played a pivotal role in both preoperative diagnosis and postoperative volume assessment.

## References

[1] International Society of Aesthetic Plastic Surgery (ISAPS). International Survey on Aesthetic/Cosmetic Procedures Performed in 2023. International Society of Aesthetic Plastic Surgery; 2023.

[2] FDA. Silicone Gel–Filled Breast Implants—Rupture Rates. U.S. Food and Drug Administration; 2022.

[3] Heden P, Nava MB, van Tetering JPM, et al Prevalence of rupture in Inamed silicone breast implants. Plast Reconstr Surg. 2006;118:303–308. 16874191 10.1097/01.prs.0000233471.58039.30

[4] U.S. Food and Drug Administration. Breast Implant Guidelines. Accessed May 9, 2026. https://www.fda.gov/medical-devices/breast-implant/

[5] Juanpere S, Perez E, Huc O, Motos N, Pont J, Pedraza S. Imaging of breast implants—a pictorial review. Insights Imaging. 2011;2:653–670. doi: 10.1007/s13244-011-0122-3 22347984 PMC3259319

[6] Goldammer F, Pinsolle V, Dissaux C, Pélissier P. Accuracy of mammography, sonography and magnetic resonance imaging for detecting silicone breast implant ruptures: a retrospective observational study of 367 cases. Ann Chir Plast Esthet. 2021;66:25–41. doi: 10.1016/j.anplas.2020.09.001 32988663

[7] Nahabedian MY. Autologous breast reconstruction: TRAM/DIEP flap outcomes. Clin Plast Surg. 2007;34:105–116. doi: 10.1016/j.cps.2006.10.01117307075

[8] Coleman SR. Augmentation of the breast and tuberous breast. In: Coleman SR, Mazzola RF, Pu LLQ, eds. Fat Injection: From Filling to Regeneration, 2nd ed. Thieme Medical Publishers; 2018:653–694.

[9] Brown MH, Shenker R, Silver SA. Cohesive silicone gel breast implants in aesthetic and reconstructive breast surgery. Plast Reconstr Surg. 2005;116:768–779. doi: 10.1097/01.PRS.0000179739.45032.35 16141814

[10] Ørholt M, Weltz TK, Hemmingsen MN, et al. Long-term volume retention of breast augmentation with fat grafting depends on weight changes: a 3-year prospective magnetic resonance imaging study. Plast Reconstr Surg. 2025;155:947–954. doi: 10.1097/PRS.0000000000011841 39465661

[11] Groen JW, Negenborn VL, Twisk DJ, et al Autologous fat grafting in cosmetic breast augmentation: a systematic review on radiological safety, complications, volume retention, and patient/surgeon satisfaction. Aesthetic Plast Surg. 2016;36:993–1007. doi: 10.1093/asj/sjw048 27329661

[12] Khouri RK Jr, Khouri RE, Lujan-Hernandez JR, et al Diffusion and perfusion: the keys to fat grafting. Plast Reconstr Surg Global Open. 2014;2:e220. doi: 10.1097/GOX.0000000000000183 PMC4229279 25426403

[13] Lin Y, et al Breast augmentation with autologous fat: 3D imaging evaluation of retention after Coleman technique. J Cosmet Dermatol. 2022;21:1524–1530. doi: 10.1111/jocd.14234

[14] Kaplan EL, Meier P. Nonparametric estimation from incomplete observations. J Am Stat Assoc. 1958;53:457–481. doi: 10.1080/01621459.1958.10501452

[15] Largo RD, Tchang LAH, Mele V, et al Efficacy, safety and complications of autologous fat grafting to healthy breast tissue: a systematic review. J Plast Reconstr Aesthet Surg. 2014;67:437–448. doi: 10.1016/j.bjps.2013.11.011 24394754

[16] Tissiani LA, Alonso N, et al High long-term retention of cell-assisted lipotransfer in TRAM flap reconstruction. Microsurgery. 2020;40:258–262. doi: 10.1002/micr.3044131328294

[17] Pusic AL, et al Development of a new patient-reported outcome measure for breast surgery: the BREAST-Q. Plast Reconstr Surg. 2009;124:345–353. doi: 10.1097/PRS.0b013e3181aee807 19644246

[18] Razzano S, Taylor R, Schonauer F, Figus A. How to assess the volume of a DIEP flap using a free online calculator: the DIEP V method. J Plast Reconstr Aesthet Surg. 2018;71:1476–1478. doi: 10.1016/j.bjps.2018.04.029 30104142

[19] Zingaretti N, Fasano D, Baruffaldi Preis, FW, et al Suspected breast implant rupture: our experience, recommendations on its management and a proposal for a model of informed consent. Eur J Plast Surg. 2019;43:569–576. doi: 10.1007/s00238-019-01610-1

[20] Lindenblatt N, Helbich T, Deutinger M, et al Correlation between MRI results and intraoperative findings in patients with silicone breast implants. Int J Womens Health. 2014;6:703–709. doi: 10.2147/IJWH.S58493 25114595 PMC4124066

[21] Ikeda DM, Borofsky HB, Herfkens RJ, et al Silicone breast implant rupture: pitfalls of magnetic resonance imaging and relative efficacies of magnetic resonance, mammography, and ultrasound. Plast Reconstr Surg. 1999;104:2054–2062. doi: 10.1097/00006534-199912000-00016 11149768

[22] Matros E, Albornoz CR, Razdan SN, et al Cost-effectiveness of implants versus autologous perforator flaps using the BREAST-Q. Plast Reconstr Surg. 2015;135:937–946. doi: 10.1097/PRS.0000000000001122 25517411

[23] Alderman AK, et al Complications in postmastectomy breast reconstruction. Plast Reconstr Surg. 2002;109:2265–2272. doi: 10.1097/00006534-200206000-00011 12045548

[24] Becker H, et al Long-term results of implant-based reconstruction: a 20-year review. Aesthetic Surg J. 2020;40:NP277–NP286. doi: 10.1093/asj/sjaa001

[25] McCarthy CM, et al Patient satisfaction in autologous reconstruction using BREAST-Q. Ann Plast Surg. 2010;64:573–578. doi: 10.1097/SAP.0b013e3181c863b0

